# Progress in Surgery

**Published:** 1897-09-18

**Authors:** 


### Progress in Surgery.

### DISEASES OF THE ŒSOPHAGUS AND STOMACH.

**Cicatricial Stricture of the Œsophagus.**—A case of cicatricial stricture near the cardia, the result of swallowing potash lye five years previously, was recorded by F. Petersen[1] (Kiel). Gastrostomy two months afterwards caused the patient to regain his former weight, but attempts at dilatation of the stricture proved futile. Four years afterwards Krause's operation was undertaken, and the stricture was dilated from below. The patient swallowed a silk thread, keeping one end in the mouth, while the other end was made secure by filling the stomach with water and allowing it to escape rapidly, then attaching to it an ivory dilator. To this another thread was again attached with a larger dilator, and so on. In a week a No. 28 had been drawn through. The dilatation was then continued by sounds from above until the patient could himself pass No. 50. He then demonstrated before the German Surgical Congress the facility with which he could drink a glass of beer. In three cases where the stomach could not be brought out of the abdominal wound Fischer[2] fixed it to the abdomen, and three days afterwards fed the patient with a syringe and canula passed obliquely into the viscus. By repetition of the injections at the same spot, and in the same direction, a valvular fistula was formed, which ultimately admitted a catheter.

**Foreign Bodies in the Œsophagus.**—Many of these cases require the removal of the foreign body by the aid of an operation on the stomach. White[3] reported a young child who swallowed an irregular toy called a "jack-stone," the position of which was shown by a skiagram. Gastrotomy was performed 12 days afterwards, and the toy removed by the mouth by a piece of gauze with cord attached, passed upwards through the œsophagus. Wood, of Philadelphia,[4] states an almost identical case with a similar toy, and successful result. Several cases requiring gastrostomy are detailed by W. Koerte (Berlin),[5] such as a bronze nut lodged in a stricture of the œsophagus, a screw, an anchor; also a plate of false teeth, which was supposed to have been pushed down into the stomach, but P.M. gangrene of the lung was found, and a false passage behind the œsophagus.

**Operations on the Stomach.**—Mikulicz (Breslau)[6] has collected 103 cases, and tabulated the results, with a general mortality of about 23 per cent. in selected cases, and a special mortality of 22 per cent. for cases of carcinoma, 8 per cent. for non-carcinomatous ones, and 83 per cent. for severe complications of gastric ulcer. Shock was the chief cause of death, and pneumonia came next in frequency. The average duration of life after gastrostomy, gastro-enterostomy, and pylorectomy for cancer was respectively 5, 7½, and 15 months.

**Cancer of the Œsophagus — Gastrostomy.**—Gastrostomy is now so frequently performed that comparatively few of the cases, even successful ones, are published. Mr. C. B. Lockwood[7] described a case which had been treated by Symonds' tubes, but as the man swallowed the "safety-string," gastrostomy had to be performed for a double purpose. The tube, however, only came to light at the post-mortem three weeks afterwards. Symonds' tubes also failed in the case mentioned by Mr. Pearce Gould,[8] in which he subsequently did Kocher's operation, *i.e.*, bringing the stomach through a vertical incision through the rectus, passing it under a bridge of skin raised for the purpose, and fixing it to the edges of a second incision over the costal margin. The first skin incision is then closed. The idea of a reliable sphincter in this case proved fallacious, as in most cases when the operation is done through the rectus. Dr. Willy Mayer[9] brought before the notice of the New York Surgical Society a case of gastrostomy by Kader's method, in which a rubber tube is first of all fixed in the stomach, and then hemmed in by bringing two separate layers of the sero-muscular coat together around the tube. Then the parietal peritoneum and transversalis fascia are fixed to the stomach, and the operation completed extra-peritoneally. Mr. Leonard Bidwell[10] argues in favour of peritoneal union of stomach to parietes. He, too, mentions a case in which gastrostomy was needed owing to the patient having bitten through the silk of a Symonds' tube.

**Gastric Ulcer, Perforation, Hæmorrhage.**—With regard to the surgery of gastric ulcer, some surgeons excise the ulcer before union of the stomach wall; others do not. Partial gastrectomy is occasionally performed for simple chronic ulcer, but this treatment is not in general favour. The cases of perforated or bleeding gastric ulcer cured by operation are now numerous, and each success represents a life saved.

In 1896 Mr. Littlewood[11] had two successful cases, the operations being done six and nine hours respectively after perforation, and in both cases the ulcers were excised. He attributed much of his success to the early diagnosis. Dr. St. Thomas Clarke, of Leicester,[12] reported a successful case; and Mr. Morse, of Norwich,[13] recorded three cases, two of which were successful; the fatal case was not operated on till after the lapse of twenty-four hours. Mr. Quarry Silcock[14] had a case which had gone twenty-four hours, which, however, proved successful, showing that a perforated gastric ulcer is "never too late to mend." Nevertheless, although the affection is so fatal and the operative results so brilliant, cases of perforated gastric ulcer do occasionally get well without operation; of this there can be no doubt, although the chances are very much against such an issue. Dr. Claude Taylor and Dr. E. A. Searle[15] recently brought under notice two such cases of recovery without operation. The *Lancet* of Dec. 5, 1896, gives an interesting clinical lecture by Mr. Arthur Barker on seven cases with three recoveries, with valuable observations based upon their behaviour after various details of treatment. The ulcers were not cut out, and Mr. Barker states that he prefers dry sponging of the peritoneum to flushing. The factors most favourable to successful operation are (1) early diagnosis and treatment, (2) situation of the ulcer on the anterior wall of the stomach, when it is found at once, sutured with ease, and does not involve the lesser cavity of the peritoneum. With regard to operation in cases of repeated hæmatemesis from gastric ulcer, it has already been successfully performed, and claims a place in surgery.

**Strictures of Pylorus—Non-Malignant.** — Loreta's operation of stretching the pyloric orifice from within through an incision in the stomach has fallen largely into disuse, and pyloroplasty, in which a longitudinal incision through the pylorus is sewn up transversely, seems to obtain more favour. Mr. Rutherford Morison[16] performed pyloroplasty in four cases, and states " no case in abdominal surgery have given me more satisfaction than these. In each case recovery from the operation has been without incident, and the first and third cases have been veritable resurrections." Mr. Mayo Robson[17] also remarks that pyloroplasty is not only easy and safe, but permanently beneficial. He uses one of his bone bobbins in addition to suturing the longitudinal incision in a transverse way, and recommends two continuous sutures, one of catgut for the mucous coat, and one of silk for the serous. Allied to stricture of the pylorus is the rarer condition of kinking of the pylorus produced by a suppurating gall-bladder.[18]

**Cancer of the Stomach—Resection.**—Resection of a large portion of the stomach for cancer of the greater curvature is rarely done; but a remarkable case occurred in the practice of Madyl,[19] in which a piece of stomach over seven inches square was removed from the greater curvature, together with some enlarged glands, and the patient remained alive and well at the end of six years. Microscopically the growth appeared to be a carcinoma, but it should be noted that the report states that the mucous membrane was intact. There must, therefore, be an element of doubt. Resection of the pylorus for carcinoma has largely given way to gastro-jejunostomy, owing to the mortality of the former, and few cases are nowadays recorded.

**Foreign Bodies in the Stomach.**—Extraordinary accumulations are to be seen in most museums of articles swallowed by the insane. Dr. Fricker[20] gives an example of a woman, once hysterical, but now melancholic after repeated child-bearings, who had enough in her stomach to furnish a curiosity shop—a key, a silver tea-spoon, a metal tea-spoon, a fork, two tacks, two half hair-pins, two pieces of glass, a window-hook, a steel pen, nine sewing needles, a piece of black-lead, a shoe-button, a grape stone, two pieces of tin, and a crochet-hook. Her stock-in-trade was confiscated by the surgeon, and she made a good recovery.

**Gastro-enterostomy.** — At the Clinical Society of London Mr. Ernest Lane[21] read notes of two cases performed by means of Murphy's button, one of which cases, some three months after operation, still retained the Murphy button. Mr. Barker was of opinion that the operation could be done as well with sutures or any other mechanical contrivance.

**Hour-glass Contraction of the Stomach.**—This rare condition was successfully treated by Lauenstein[22] after many years of palliative measures by the formation of an anastomosis between the cardiac and pyloric sacs.

[1] Verhandlungen der Deutscher Gesellschaft für Chirurgie XXIV., Kongress, 1895. [2] Ditto. [3] Univ. Med. Mag., June, 1896. [4] Ditto, October, 1896. [5] Verhandlungen der Deutscher Gesellschaft für Chirurgie, XXIV., Kongress, 1895. [6] Ditto. [7] Lancet, April 3rd, 1897. [8] Lancet, Sep. 19th, 1896. [9] Annals of Surgery, Jan., 1897 (illustrated). [10] B. M. J., Oct. 24th, 1896. [11] Lancet, Oct. 31st, 1896. [12] Lancet, Mar. 20th, 1897. [13] B. M. J., Feb. 13th, 1897. [14] Lancet, April 24th, 1897. [15] Lancet, Dec. 19th, 1896. [16] Lancet, Oct. 24th, 1896. [17] Edinburgh Med. Jour., Jan., 1897. [18] B. M. J., Jan. 23rd, 1897. [19] B. M. J. Epitome, Oct., 17th, 1896. [20] Deutsche Medicinische Wochenschrift, No. 4, 1897. [21] B. M. J., Feb. 20th, 1897. [22] Münch Med. Woch, May, 1896.

### GENERAL SURGERY.

(*Continued from page* 412.)

**Lower Extremity.**—Dr. Royal Whitman[27] believes that fracture of the neck of the femur in childhood occurs more frequently than is generally supposed, because of failure to recognise the true nature of the injury. In 1890 he first brought forward a case of this kind, four others in 1892, and since then he has seen five more cases. In 1890 the diagnosis of fracture of the neck of the femur was made largely by exclusion, but to-day could be confirmed by skiagraph. In nearly all instances the patients were brought by the parents, with the supposition that they were suffering from hip-joint disease. The subject has not yet been considered in the text-books separate from the same accident in adults. Freeman considers the subject under four heads—(1) The intermediate diagnosis and effects; (2) differential diagnosis from hip disease during recovery; (3) diagnosis between fracture and epiphysial separation; (4) remote effects of fracture. All these points he has embodied in a recent article in " The Annals of Surgery,"[28] where he has also added photographs and diagrams of clinical cases, and a skiagraph of one case. An ununited fracture of the upper third of the femur in a man aged 48 was lately operated upon by Stephen Paget.[29] After many weeks of treatment there had not been the least attempt at union. Three fragments were found to exist—for a long thin shell of bone had been broken off the inner aspect of the shaft, and lay to the inner side of the lower fragment; the fragments were as smooth and devoid of callus as if the fracture had only just occurred. The third fragment was lashed to the lower by very thick silver wire passed round them, and the upper to the lower fragment wired by drilling them. The result was successful; the patient has now abundance of callus and firm union. Haudek,[30] of Vienna, recently brought forward a child who had fractured the middle third of its femur three weeks before, and had been treated by the application of a Hessing fracture splint apparatus, viz., a combination of splints, pelvic band, and foot piece, made over a plaster cast of the limb in extension, applied over a tricot stocking and a bandage wound from the toes to prevent œdema. The child could stand and walk, if led, without pain, immediately after the light apparatus was applied, and in a day or two could walk alone. The joints were left free from the first. A skiagraph of a case of fractured femur close above the knee-joint, which occurred six months previously, is given by Surgeon-Colonel W. F. Stevenson and Surgeon-Major Whitehead.[31] The end of the upper fragment appeared jagged and sharply defined as in a recent fracture. The man, however, could walk without any sign of lameness or pain in the part. That the bone was in fact as sharp as it appeared could not be believed. It is probable that the X rays had traversed the more recent callus, which really rounded off the end, without throwing a shadow on the plate, while the older bone is clearly defined. Another skiagraph published by Dr. De Forest Willard[32] reveals a longitudinal fracture of the tibia, with fracture of the malleolus and fracture of the fibula (three inches above the ankle joint). The accident had occurred seven months previously to a blacksmith. The foot was still everted, as in Potts' fracture, and walking caused great pain so that the patient was totally disabled. Dr. Joseph Griffiths[33] puts on record an interesting example of spontaneous fracture of the right tibia and fibula in a case of locomotor ataxia. The patient was a tailor, aged forty-six. Six days after admission an incision was made over the fracture and the broken ends examined. There was no trace of malignant disease, but the tibia was harder and more brittle than natural. The wound was closed, and the limb fixed in plaster of Paris. Union in due time became complete. The ambulatory treatment of fractures of the leg, as employed in the Roosevelt Hospital, New York, since 1888, was introduced by Dr. J. M. Woodbury, according to Dr. J. P. Fiske.[34] It possesses certain new principles. After the bones were in place a plaster splint was applied from the toes to near the knee, and the patient was allowed to walk upon the limb from the second or even the first day in some instances, but was occasionally kept in bed for ten days. A roller bandage was the only padding beneath the splint. Some starch was added to one of the three layers of plaster bandage, to give slight elasticity. The foot was at a right angle to the leg. Thus applied, the upper part of the cast supported the greater part of the weight of the body. While sometimes the patient was allowed to walk at once, the rule was to keep him in the horizontal position for twenty-four hours, and if at the end of that time there was no pain he was directed to walk. The same rule applied to compound fractures, if there was no infection. The cast was removed if it weakened. The ambulant treatment has been employed by Martin[35] in upwards of 30 cases of fractures of the leg with good results. There were two cases in which the results were not thoroughly satisfactory, but in these the surgeon did not apply the dressing personally. One was an instance of Potts' fracture. The deformity was not greater than is often noticed after treatment by the fracture box. The other was one of compound fracture, for which the patient was operated on. Martin has never used the dressing for fractures above the knee, and, with the exception of the last case, all have been simple fractures. The Germans apply the ambulant dressing not only to fractures of the leg, but also for simple and compound fractures of the thigh. The way in which A. G. Miller[36] employs massage in the treament of simple fracture of the leg is as follows: In a case of simple fracture, whether of both bones or of the fibula only, the limb is put up in the usual way—most frequently in the box-splint, so called, made with a sheet and two pieces of wood. Next day the massage is commenced, unless bullæ and excoriations of the skin exist. The masseur (*i.e.*, the surgeon, house surgeon, dresser, or nurse) undoes the slip knots of the splint, folds back the wooden sides, removes all padding carefully, and then, steadying the leg with one hand, proceeds to stroke the thigh gently upwards (effleurage). This is continued for a few minutes, going downwards till the fracture is reached. Firmer manipulation of the leg (pétussage) is then employed with the fingers, especially if there should be much effused blood, the fracture being steadied. Lastly, the limb is very carefully raised from the splint, and the ankle-joint gently moved, the fracture being supported with great caution. The limb is then replaced in the splint and adjusted. The massage lasts for five or ten minutes, and is repeated daily, or only every two or three days, or longer. It may be commenced immediately after the injury, or in a day or two, or a week, but the earlier the better. Miller epitomises its advantages, as (1) swelling and effusion are got rid of more quickly; (2) stiffening of the ankle and knee joints by adhesions is prevented; (3) union is facilitated; (4) time is saved, which is a very important point for the patients.

[27] N. Y. Medical Record, Feb. 20, 1897, p. 280. [28] Annals of Surgery, June, 1897, Part 54, p. 673. [29] Clinical Journal, April 28, 187, p. 9. [30] Wien Klin, Rundschall, Jan. 17, 1897; and Clinical Journal, March 31, 1897. [31] Lancet, March 20, 1897. [32] Annals of Surgery, June, 1897, p. 763. [33] Brit. Med. Jour., April 24, 1897. [34] Medical Chronicle, April, 1897, p. 47; Med. News, Feb. 13, 1897; and N. Y. Med. Record, Feb. 6, 1897, p. 204. [35] Philadelphia Polyclinic, Jan. 2, 1897; and Therapeutic Gazette, March 15, 1897. [36] Practitioner, March, 1897, p. 264.

